# Dual high-Q Fano resonances metasurfaces excited by asymmetric dielectric rods for refractive index sensing

**DOI:** 10.1515/nanoph-2023-0840

**Published:** 2024-02-07

**Authors:** Tianyu Wang, Siqi Liu, Jiahang Zhang, Liang Xu, Mingyu Yang, Ding Ma, Sijia Jiang, Qingbin Jiao, Xin Tan

**Affiliations:** Changchun Institute of Optics, Fine Mechanics and Physics, Chinese Academy of Sciences, Changchun, Jinlin 130033, China; University of Chinese Academy of Sciences, Beijing 100049, China

**Keywords:** all-dielectric metasurface, Fano resonance, BIC, refractive index sensor

## Abstract

The metasurface refractive index sensor has a high degree of tunability and flexibility, providing excellent performance for high precision refractive index sensing applications. The metasurface absorber with metallic structure has been hindered in further sensor applications due to the inherent Ohmic loss of the metallic material. In this study, a dual nanorod metasurface structure based on semiconductor Si was designed, introducing a symmetry-breaking structure to excite dual ultra-narrow q-BIC resonance peaks with Fano line shapes. Both peaks are located in the near-infrared region, and multipole analysis shows that this strong field enhancement effect is induced by a magnetic dipole. Experimental results demonstrate the potential of this sensor to provide dual-channel detection while achieving high sensitivity and high Q-factor. We believe that this device exhibits outstanding performance and high practicality, providing a reference for the development and application of biological and environmental sensors.

## 1 Introduction

Refractive index detection is of significant importance in the fields of material identification, quality control, chemical analysis, optical design, and environmental monitoring. In recent years, optical refractive index sensors based on metamaterials have attracted increasing attention due to their more integrated, stronger light-capturing capabilities, and the ability to detect biomolecules without labeling [1]–[3]. Metasurfaces are artificially arranged periodic subwavelength microstructures that can be considered as a two-dimensional form of metamaterials. Researchers have theoretically verified and prepared a wide range of metasurface devices, such as metalens [4], filters [5], sensors [6]–[9], etc., based on the unique optical properties of metasurfaces. By controlling the resonance modes of metasurface structures, a higher localised field enhancement effect can be generated near the metasurface devices, which is beneficial for achieving high sensitivity and precision sensors.

However, the oscillation of free electrons in metallic metasurfaces leads to significant radiation losses [10], and the quality factors of resonant dips are usually low, limiting the application of metal-based plasmonic resonance micro-nano devices in nanophotonics. Dielectric materials based on silicon, silica, alumina, etc., can significantly reduce radiation losses and obtain higher quality factors [11]–[13]. The optical fields in an all-dielectric metasurface device are mainly confined within the device, which is beneficial for enhancing the interaction between electromagnetic waves and matter. In addition, the fabrication methods for dielectric materials are compatible with MEMS processes, allowing for large-scale mass production.

Fano resonance is generated by the interference of a broadened bright mode and a narrow-band dark mode [14], [15], resulting in a sharp spectral line characteristic that has gradually delved into the realm of metasurface absorber design. The excitation of high-Q factor Fano resonance using bound states in the continuum (BIC) has been an effective method. BIC is a special quantum state that exists within the background of a continuous spectrum and has an infinite lifetime [16], [17]. This optical wave mode is unable to radiate energy within a prohibited frequency range, leading to the localization of light energy in a specific region. By breaking the symmetry of local space, such as structural asymmetry, material asymmetry, and polarization asymmetry, BIC can be transformed into quasi-BIC (q-BIC), thereby obtaining ultra-narrow linewidth spectral lines [18].

Previous studies have investigated designs that utilize q-BIC to excite Fano resonance in all-dielectric materials, including diameter-asymmetric nanodisks [19], length-thickness asymmetric nanorods [20]–[22] and optics with high field enhancement factors [23]–[26], and nano-clusters [27]. Nevertheless, these methods have not been able to achieve high quality factors, high sensitivity, and multiple resonance dips simultaneously. This paper presents a transmissive metasurface sensing structure composed of asymmetric dielectric nanorods. By controlling the structural parameters of Si nanorods, dual Fano resonance dips were excited in the near-infrared (<1700 nm) region. Theoretical simulations proved that the maximum Q factors were 7750 and 2850, with the minimum transmittance value close to 0. The multipole decomposition revealed that the two resonant primary electromagnetic sources are primarily magnetoelectric dipole (MD) excited toroidal dipole (TD). Additionally, the effect of metasurface structure period and nanorod thickness on the resonance intensity has been investigated. Finally, the device was created and its functionality was evaluated using equipment including electron beam lithography. The device achieved a high sensitivity in detecting refractive index, which offers significant guidance towards the advancement of all-dielectric metasurface refractive index sensor devices.

## 2 Metasurface design and analysis

**Figure 1:**
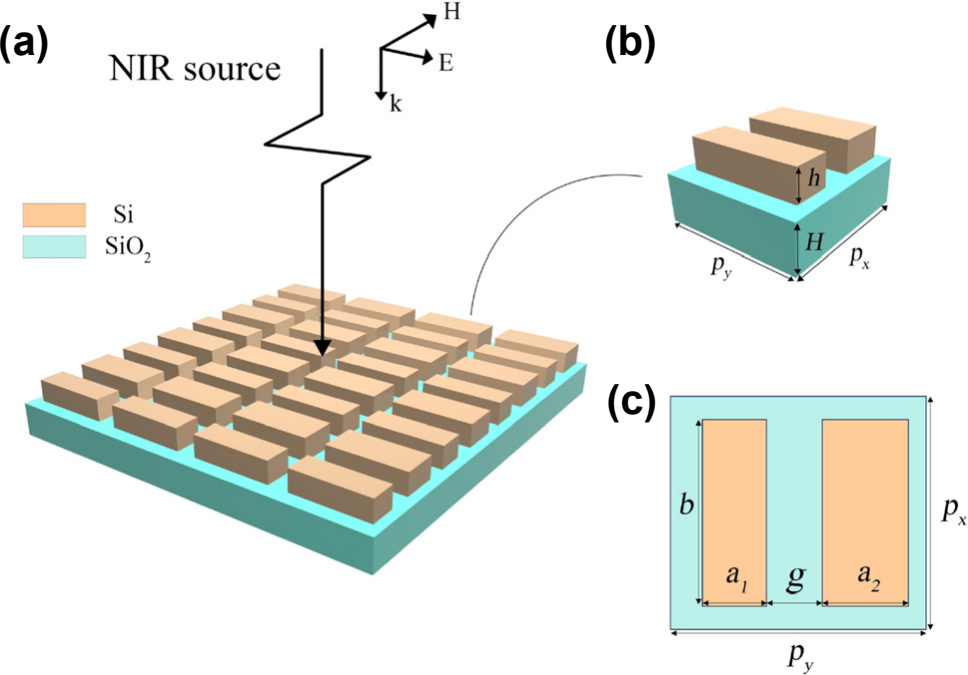
Structure and principles of metasurface sensing systems based on asymmetric nanorods. (a) Structural diagram of the designed metasurface device. (b) Structural diagram of a single cycle. (c) Top view of a single periodic structure.

The designed metasurface structure as shown in Figure 1. The device consists of silicon nano-rods etched on a quartz substrate, with each pair of differently-sized nano-rods forming a metasurface period. Figure 1(a) illustrates a schematic of the metasurface device. Near-infrared source is incident from above the device with a wave vector in the *Z* axes, electric field direction in the *Y* axes, and magnetic field direction in the *X* axes. Figure 1(b) represents the structure of each metasurface unit, while Figure 1(c) shows a top view. Here, *p*_*x*_ and *p*_*y*_ denote the lengths of the metasurface unit extending in the *X* and *Y* axes; *h* and *H* are the thicknesses of the silicon nano-pillars and the quartz substrate; *a*_1_ and *a*_2_ represent the widths of the two asymmetric nano-rods; *b* is the length of the nano-rods, which are equal in this structure; and finally, *g* represents the gap between the nano-rods.

In order to analyze the structure, we performed simulations using commercial software packages: Lumerical FDTD, which utilizes the finite-difference time-domain (FDTD) algorithm. The method involves setting perfect match layers (PML) in the *Z* axes and periodic boundary conditions in the *X* and *Y* axes. The grid size is less than 1/20*λ*, and the simulation time for FDTD is set to be greater than 80,000 fs to guarantee accuracy. Refractive index data on Si and quartz used during the simulation process are obtained from Li [28] and Tan [29].

### 2.1 Spectral and near-field properties

**Figure 2:**
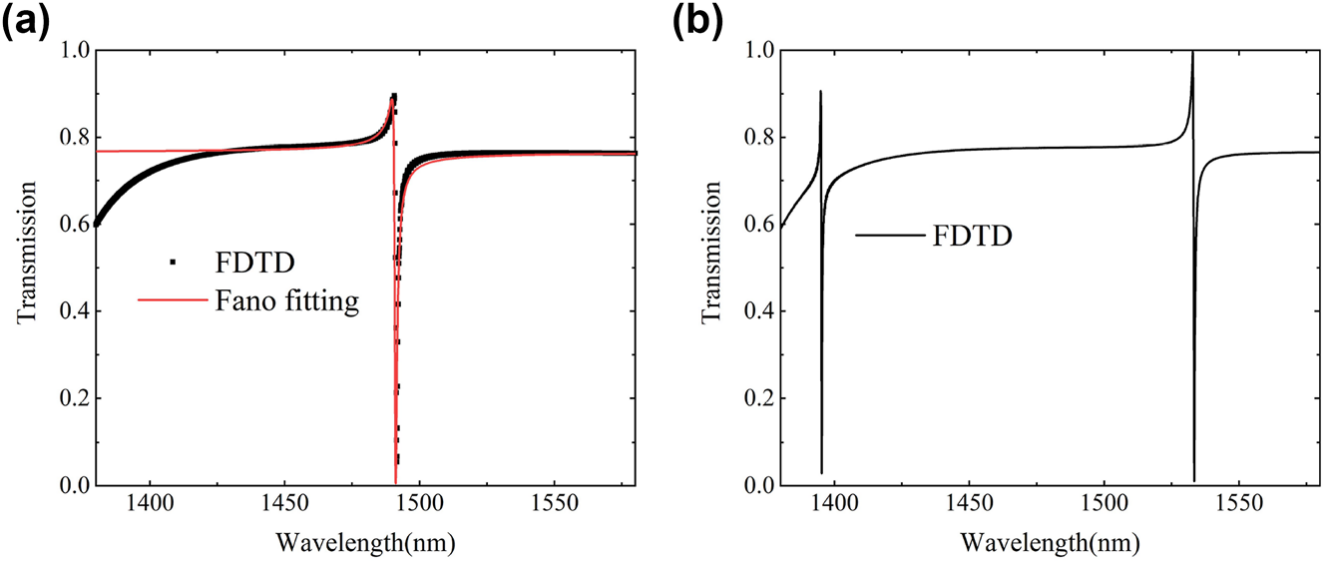
Spectral transmission curves for symmetric and asymmetric structures. (a) Transmission curve and Fano fitting results (red curve) when *δ* = 0. (b) Transmission curve at *δ* = 80 nm.

The structural simulation analysis was carried out using the above method. The initial values of the structural parameters *p*_*x*_, *p*_*y*_, *a*_1_, *a*_2_, *b*, *g*, *h*, and *H* are 950 nm, 900 nm, 240 nm, 320 nm, 720 nm, 210 nm, 200 nm and 1000 nm. Moreover, the asymmetry parameter is defined as *δ* = *a*_2_ − *a*_1_ = 80 nm. For comparison, simulations were also conducted on a symmetric nanorod structure, where *a*_1_ = *a*_2_ = 280 nm, which means *δ* = 0, while keeping the other parameters constant. Figure 2(a) shows the transmission spectrum of the symmetric structure metasurface, exhibiting a Fano resonance dip at 1491 nm. Figure 2(b) presents the transmission spectrum of nanorod structures with different widths. Introducing the asymmetry parameter results in additional transmission dips at 1395 nm and 1538 nm, with a full width at half maximum (FWHM) of approximately 0.18 nm and 0.5 nm. The trends obtained from the two algorithms are generally consistent, providing additional confirmation of the feasibility of this structure and laying the groundwork for the preparation of subsequent devices.

The Fano resonance curve can be described by the following formula [30], [31]

$$T\left(\omega \right)=T_{0}+A_{0}\frac{{\left[q+2\left(\omega -\omega_{0}\right)/\Gamma \right]}^{2}}{1+{\left[2\left(\omega -\omega_{0}\right)/\Gamma \right]}^{2}}$$

Among them, *ω*_0_ represents the resonance frequency, Γ denotes the resonance dip line width, *T*_0_ stands for the background scattering parameter, *A*_0_ represents the coupling coefficient, and *q* determines the asymmetry of the resonance spectrum. In Figure 2(a), it can be observed that the simulated curve aligns well with the fitted curve. The quality factor of the resonance dip Q is obtained by dividing *ω*_0_/Γ, which yields calculated values of 7750 and 2850, respectively.

**Figure 3:**
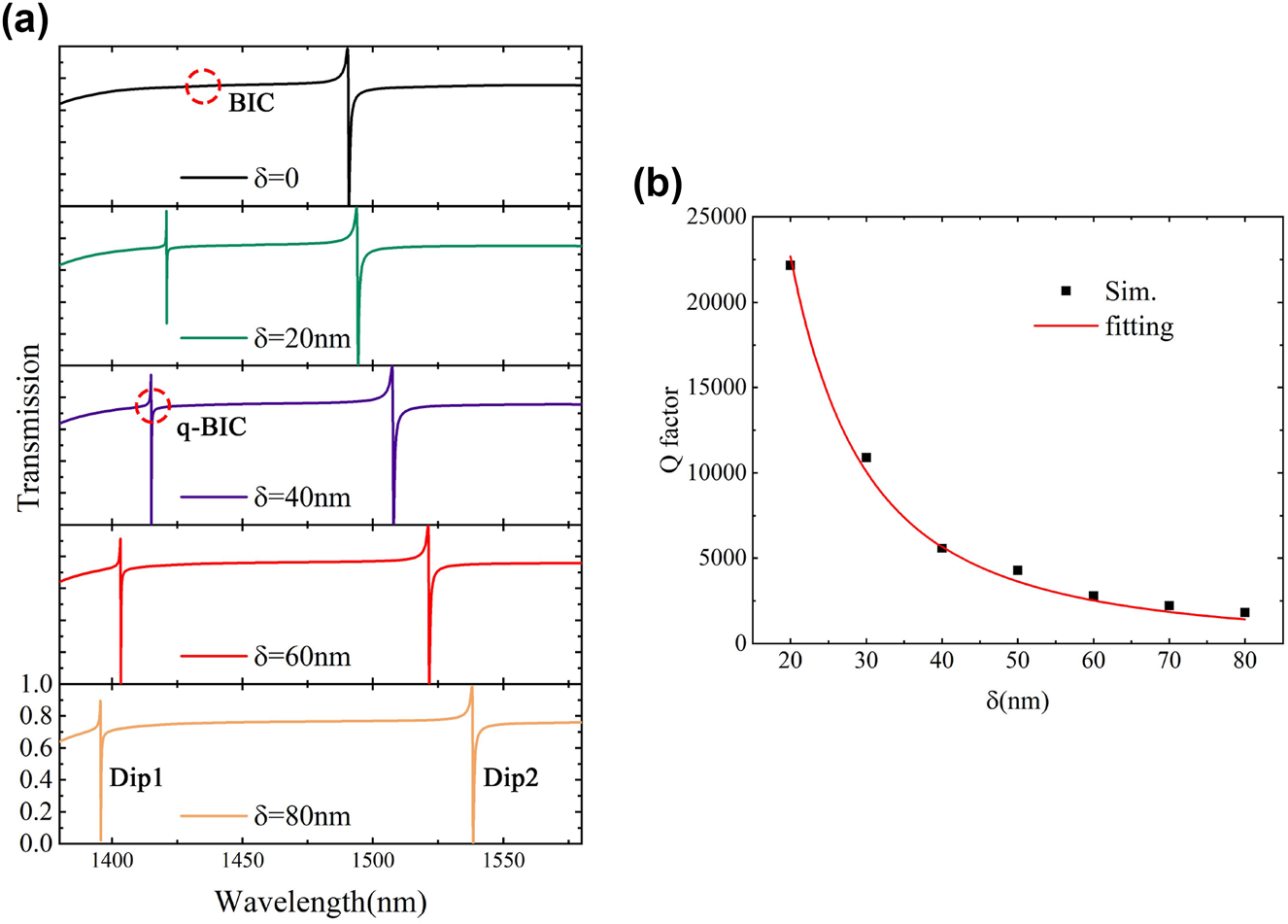
The simulation calculates the effect of asymmetry on the transmission spectrum of the metasurface. (a) The influence of different asymmetry factors *δ* on the resonant peak position and line width. (b) Simulation and power function fitting results of the relationship between *δ* and the Q factor of the Dip1 curve.

The introduction of asymmetric structure leads to the transition from BIC to q-BIC, where the energy confined in the structure is leaked out. Figure 3(a) illustrates the impact of asymmetric parameter variations on the transmission characteristics of the metasurface. When *δ* = 0, the spectrum exhibits a single resonant dip, with the linewidth at the BIC approaching infinity and thus the Q factor approaching infinity, making it indiscernible in the transmission spectrum. When *δ* ≠ 0, an additional Fano resonance dip is generated between 1390 and 1425 nm. With the increase of *δ*, more energy leaks from the localized resonance, resulting in the blue shift of the q-BIC dip and the gradual increase of the linewidth. Notably, at *δ* = 20 nm, the Q factor of the lower wavelength resonance dips can reach 22,500, which significantly surpasses those of typical metallic materials and dielectric metasurface resonators [32]–[34]. According to the BIC theory [35], [36], the Q factor should be inversely proportional to the square of the asymmetry factor, that is, *Q* = *A*/*δ* ^2^ (*A* is a constant), which is in good agreement with the simulation and fitting results in Figure 3(b). In order to achieve narrower linewidth and higher Q factors, the asymmetry parameter *δ* needs to be reduced, which also increases the difficulty of device fabrication to some extent. For subsequent analysis, *δ* was set to 80 nm.

**Figure 4:**
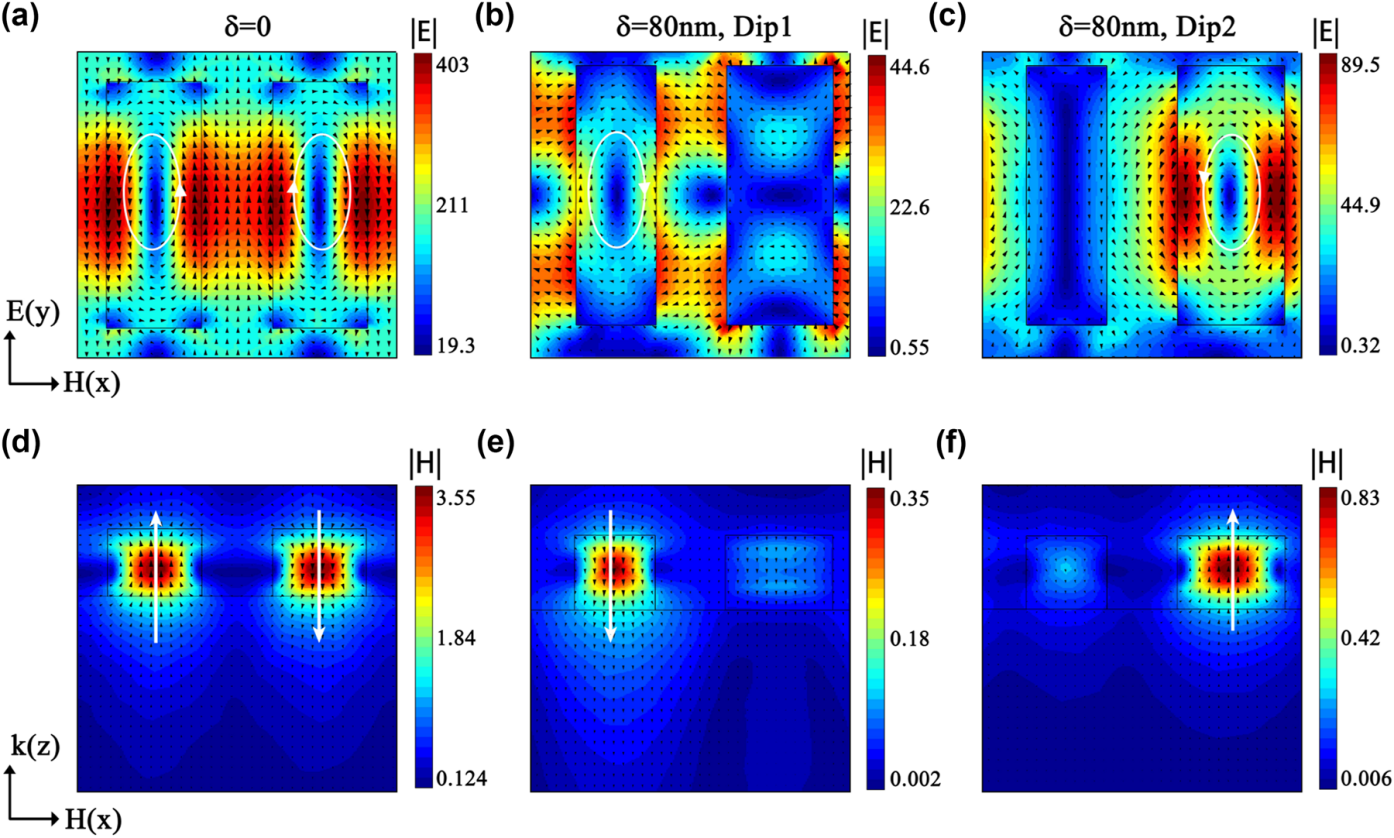
The electromagnetic field distribution in different planes of the metasurface structure at the resonant wavelength. (a) The symmetric structure exhibits the electric field |*E*| distribution in the *x–y* plane. (b), (c) The asymmetric structure shows two resonance dips with electric field |*E*| distribution in the *x–y* plane. (d)–(f) Corresponding magnetic field |*H*| distribution is shown in the *x–z* plane. The range in the *z*-direction is from −1000 nm to 500 nm.

The primary radiation source is determined by calculating the electromagnetic field distribution at the resonant position of the metasurface near-field. Additionally, the principle behind generating ultra-high Q-factor resonance is further analysed. Figure 4 shows the electromagnetic field distribution at the resonant position when *δ* = 0 and *δ* = 80 nm. Among them, (a) shows the electric field distribution in the *x–y* plane (*z* = 1/2*h* = 100 nm) of the unique resonance dip when *δ* = 0, (b) and (c) show the electric field distribution in the *x–y* plane of the resonance Dip1 with a shorter wavelength and the resonance Dip2 with a longer wavelength when *δ* = 80 nm, and (d)–(f) show the corresponding magnetic field distribution in the *x–z* plane. In the symmetrical arrangement, the electric fields’ directions on the left and right nanorod surfaces are opposite, resulting in two magnetic dipole moments (MD) with opposite directions (see Figure 4(d)). These MDs subsequently stimulate the development of a circular dipole moment (TD), leading to high Q-factor Fano resonance dips. When the nanorod width is asymmetrically introduced, the TD mode is disrupted and replaced with two MDs located on each of the nanorods. Figure 4(e) and (f) illustrates that the two MDs independently stimulate oscillations without interfering with one another.

In order to better illustrate this resonance mode, the near-field of the metasurface can be decomposed into Multipole expansion [37], [38]. The Multipole expansion mainly includes the following strongest electromagnetic sources: electric dipole (ED), magnetic dipole (MD), electric quadrupole (EQ), magnetic quadrupole (MQ), and toroidal dipole (TD). The radiation contributions of higher-order electromagnetic sources such as electromagnetic octupoles and toroidal quadrupoles are relatively small, and they are all coupled from lower-order dipoles, generally not considered. The calculation formula for multipole expansion is as follows.

(1) $$P=\frac{1}{i\omega }\int jd^{3}r$$

(2) $$M=\frac{1}{2c}\int \left(r\times j\right)d^{3}r$$

(3) $$Q_{\alpha \beta }^{\left(e\right)}=\frac{1}{2i\omega }\int \left[\left(r_{\alpha }j_{\beta }+r_{\beta }j_{\alpha }\right)-2/3\left(r\cdot j\right)\delta_{\alpha ,\beta }\right]d^{3}r$$

(4) $$Q_{\alpha \beta }^{\left(m\right)}=\frac{1}{3c}\int \left[{\left(r\times j\right)}_{\alpha }r_{\beta }+{\left(r\times j\right)}_{\beta }r_{\alpha }\right]d^{3}r$$

(5) $$T=\frac{1}{10c}\int \left[\left(r\cdot j\right)r-2r^{2}j\right]d^{3}$$

Equations (1)–(5) represent ED, MD, EQ, MQ, and TD. Here, *r* is the position vector, *c* is the speed of light, *ω* is the angular frequency of light, *x* and *y* denote the direction, and *j* is the displacement current density. When obtaining data regarding the electromagnetic field and material refractive index from simulation results, equation (6) can be used to determine the far-field radiated power of different dipoles.

(6) $$\begin{array}{ll} I & =\frac{2\omega^{4}}{3c^{3}}|P{|}^{2}+\frac{2\omega^{4}}{3c^{3}}|M{|}^{2}+\frac{\omega^{6}}{5c^{5}}|Q_{\alpha \beta }^{\left(e\right)}{|}^{2}+\frac{\omega^{6}}{20c^{5}}|Q_{\alpha \beta }^{\left(m\right)}{|}^{2}\\ & \;+\frac{2\omega^{6}}{3c^{5}}|T{|}^{2} \end{array}$$

**Figure 5:**
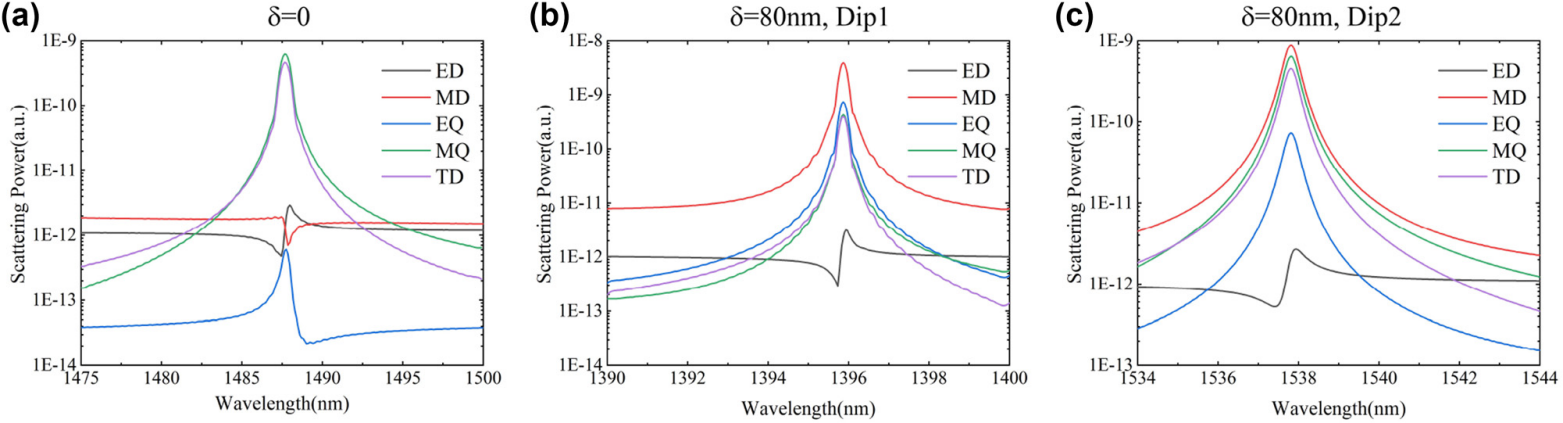
Multipole expansion of scattering modes from two structures. (a) Multipole expansion in symmetric structure. (b) And (c) are the multipolar expansion at the two dips when *δ* = 80 nm.

The multipole expansion results are shown in Figure 5. Both MQ and TD exhibit a symmetric structure that dominates the far-field radiation at resonance, and their contributions are almost equal, as evidenced in Figure 5(a). This is due to the fact that the 2D TD field distribution generates a magnetic quadrupole, whereas other electromagnetic sources are significantly suppressed [39]. When the symmetrical structure is disrupted, the two diametrically opposing MDs are stimulated at differing wavelengths, which are housed inside the two nanorod structures, releasing suppressed electromagnetic sources. The figures displayed in Figure 5(b) and (c) expound that the scattering contribution of MD hits its peak during this stage, aligning with the analysis of the mode observed from the electromagnetic field vector arrangement showcased in Figure 4. As MD collides with different modes, it leads to the creation of a resonance with a high Q-factor.

### 2.2 Influence of structure size

**Figure 6:**
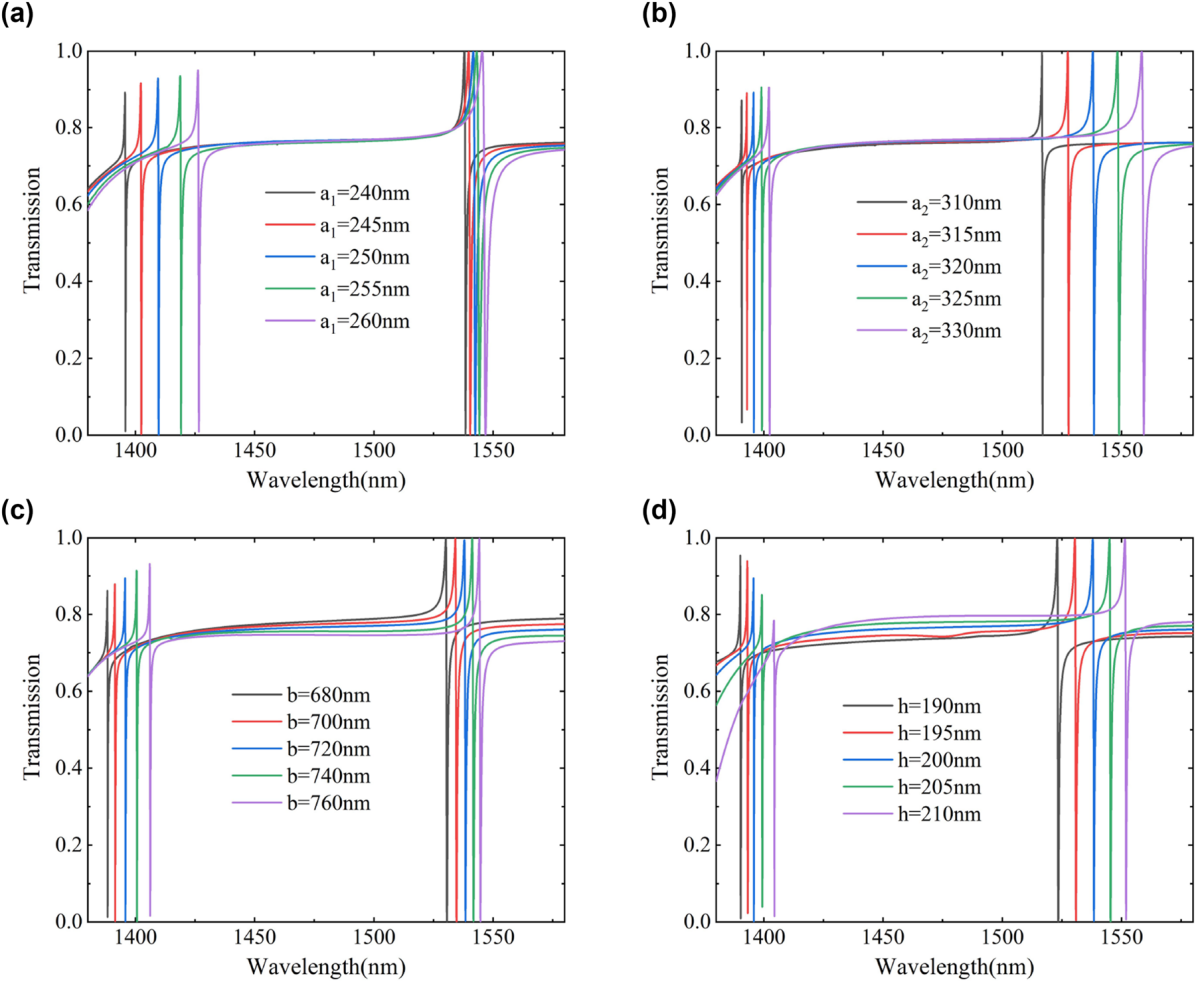
Effect of metasurface unit structural parameters on transmission spectrum. (a), (b) The variation of nanorod widths *a*_1_ and *a*_2_ affects the transmission dip. (c) The variation of nanorod length *b* affects the transmission dip. (d) The variation of nanorod height *h* affects the transmission dip.

The different sizes of metasurface structures significantly impact the performance of the sensor. Consequently, linewidth and resonance frequency of the two modes can be individually altered by adjusting geometric parameters like periodicity *p*_*x*_, *p*_*y*_, and others. To analyze the dependence of resonance frequency on the geometric parameters of the unit, the changes in the transmission curves of the metasurface were calculated when the nanorod widths *a*_1_, *a*_2_, nanorod length *b*, and nanorod height *h* varied. In all four cases mentioned above, the resonant wavelength is red-shifted. From Figure 6(a) and (b), it can be seen that the variation of *a*_1_ has a significant impact on the shorter wavelength resonant Dip1, with a noticeable red-shift of approximately 31 nm. Similarly, when *a*_2_ varies, the movement of Dip2 is more significant, approximately 43 nm. At the same time, an increase in *a*_1_ and *a*_2_ will both increase the linewidths of the two dips, while the influence of *b* and *h* is not significant (Figure 6(c) and (d)).

**Figure 7:**
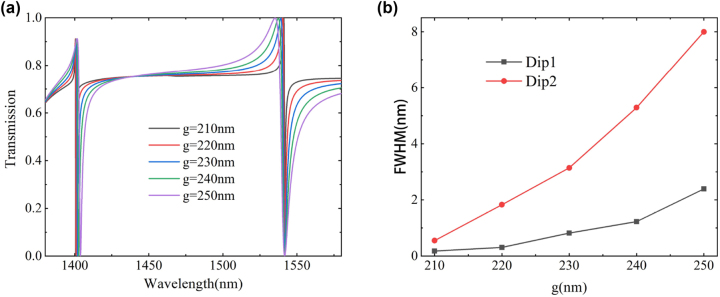
The effect of nanorod gap distance *g* on the transmission spectra of metasurface. (a) Transmission spectrum simulation results. (b) Curves of FWHM variations with *g*.

Figure 7(a) illustrates the effect of the nanorod gap distance (*g*) on the transmission spectrum of the metasurface. Changes in the gap distance have a minor impact on the position of the transmission dip, but a significant impact on the line width of the transmission dip. Figure 7(b) calculates the FWHM as a function of *g*. When *g* increases from 210 nm to 250 nm, the FWHM of Dip1 increases from 0.18 nm to 2.39 nm, and the FWHM of Dip2 increases from 0.5 nm to 8 nm. It is expected that the transmission curves will be identical for the values of the nanorod gap distance *g* and *p*_*x*_ − (*a*_1_ + *a*_2_ + *g*) due to the periodicity of the metasurface structure. Therefore, the FWHM is expected to increase followed by a decrease.

**Figure 8:**
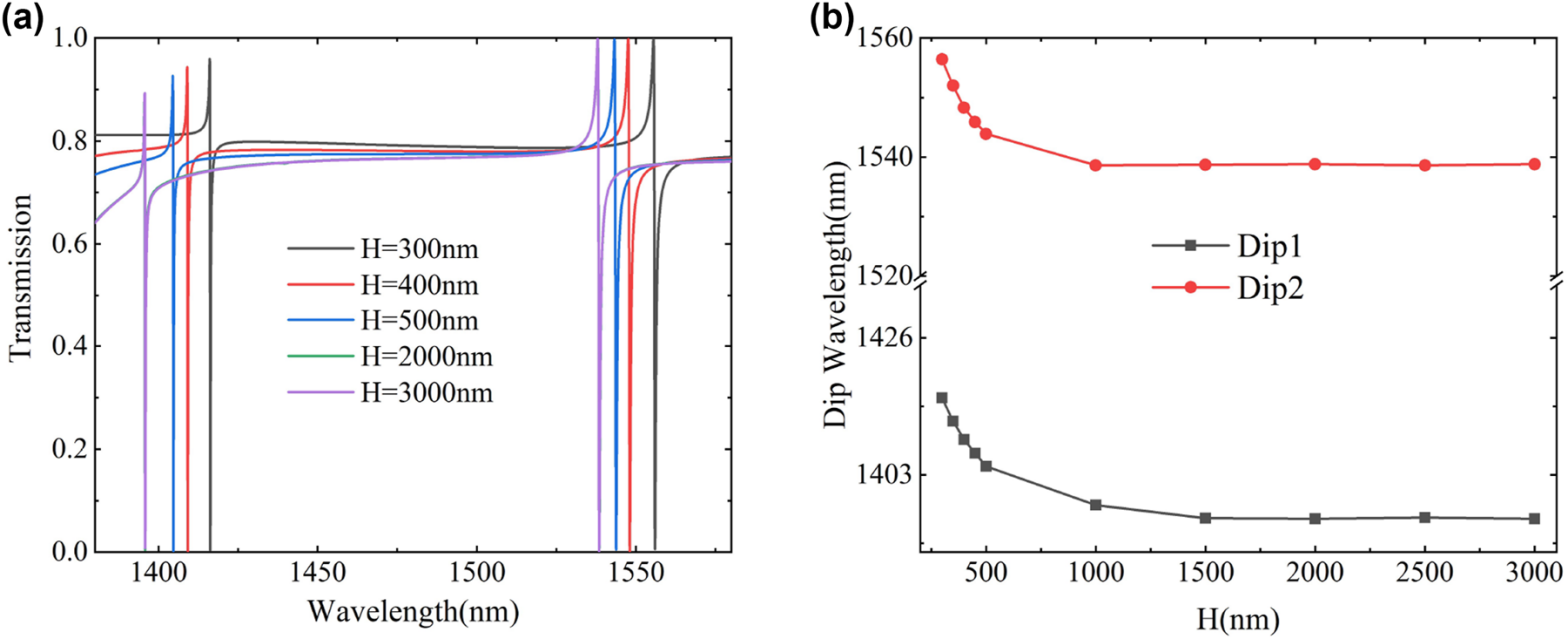
Effect of different substrate heights *H* on metasurface transmission spectra. (a) Changes in the resonance dip of the transmission spectrum. (b) The influence of *H* on the resonance dip position.

Additionally, we computed the effect of the thickness of the SiO_2_ substrate, denoted as *H*, on the resonance Dip position and transmittance (see Figure 8). Within the range of *H* < 1000 nm, the substrate thickness has a significant impact on the position of the resonance Dip and the transmittance, specifically as *H* increases, the dip is blue-shifted. At substrate thicknesses greater than 1000 nm, the transmittance dip shift gradually weakens with increasing *H*, until the curves overlap completely, indicating that the localized field of the metasurface cannot penetrate the substrate. This is further evidenced in Figure 4(d)–(f), which shows a reduction in field enhancement at *z* = −1000 nm to its minimum value.

### 2.3 Refractive index sensing performance simulation

The dip characteristics of the metasurface are closely related to the refractive index (dielectric constant *ɛ*) of the local field, so it has broad prospects in the field of refractive index sensing (medical diagnosis [40], [41], environmental detection [42], etc.). The proposed metasurface structure is simple, has high spectral contrast, and has two detection channels, while also having high process compatibility. In order to investigate the sensing performance of the designed metasurface absorber, we assume that the thickness of the test object attached above the metasurface is sufficiently large (>1000 nm), thereby excluding the influence of this factor. This is due to the fact that the thickness of the test object and the metasurface substrate can result in a shift in resonance dip, leading to a red shift followed by saturation [43]. In practical applications, the thickness of the liquid being tested often exceeds the range of the local field.

**Figure 9:**
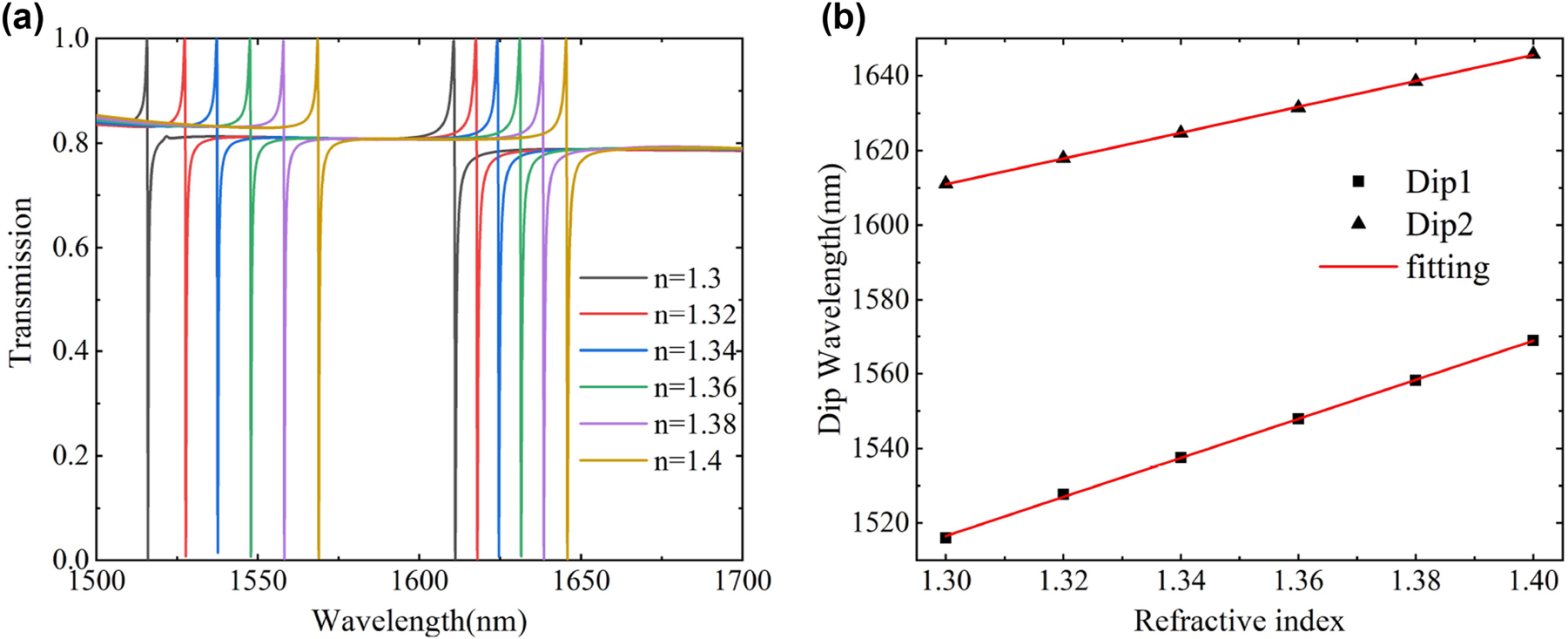
Metasurface refractive index sensing performance simulation. (a) Changes in the transmission spectrum when the background refractive index *n* increases from 1.3 to 1.4. (b) The influence of *n* on the resonant dip position and the linear fitting results (red line).

To account for this, and taking into consideration the refractive index of water (which is 1.33), we gradually increased the background refractive index *n* from 1.3 to 1.4, using a step size of 0.02. As the refractive index of the analyte increases, both dips experience a red shift, and the frequency of the shift is not the same (as shown in Figure 9). The sensitivity of the refractive index sensor can be described by *S* = Δ*λ*/Δ*n*, which Δ*λ* is the wavelength shift value of the transmission dip caused by the refractive index change. Through linear fitting, the *S* values of the two resonant dips are 528.7 nm/RIU and 347.4 nm/RIU. Additionally, the figure of merit (FOM) is an important parameter to describe the sensing performance, defined as *FOM* = *S*/*FWHM* [44]. The increase in background refractive index leads to a certain degree of reduction in FWHM. Ultimately, the calculated average FOM is 1823 RIU^−1^ and 755 RIU^−1^. We observed in the simulation that Dip1 showed better refractive index sensing performance, which I think can be explained from the electromagnetic field distribution simulation results in Figure 4. It can be observed that although Dip2 has a higher field enhancement factor than Dip1, its maximum field intensity distribution is concentrated inside the silicon nanocolumn, while the enhanced field of Dip1 is distributed around the nanocolumn (air layer, or dielectric layer). This makes the part in more direct contact with the solution to be measured, making Dip1 more sensitive to changes in the refractive index (or dielectric constant) of the solution, and therefore exhibits better sensing performance.The all-dielectric metasurface sensing structure proposed possesses high Q, high sensitivity, and high FOM attributes, with theoretical comprehensive performance parameters surpassing those of similar dielectric metasurface sensors (refer to Table 1).

**Table 1:** Performance parameters of this structure compared with other published articles.

Reference	Dip position(nm)	Q-factor	*S* (nm/RIU)	FOM(RIU^−1^)
[21]	1029	1029	125^a^	120^a^
[33]	1310, 1400, 1550	2617, 1197, 1680	220, 300, 110	400, 300, 57.9
[45]	1280, 1505	320, 780	161, 123	78,30
[46]	1670	133	453.3	56.5
[47]	967	8719	324	2465
This work (sim.)	1395, 1538	7750, 2850	528.7, 347.4	1823, 755

The data marked with ^a^are not given in the article and are calculated from other results.

## 3 Experimental results and discussion

### 3.1 Metasurface fabrication and detection methods

Based on the geometric parameters, arrangement method, and material properties of the nanorods designed above, we prepared the metasurface sensing structure in a rectangular area of 1 × 2 mm. This all-dielectric structure is fabricated using hard mask etching. Prior to fabrication, the quartz substrate was subjected to 5 min of ultrasonication with acetone, IPA, and deionized water to remove oil, dust, and other contaminants from the melted quartz surface. A 200 nm-thick *α*-Si film was deposited on the quartz substrate using magnetron sputtering. The thickness of the film determined the height of the microstructures of the resonator fabricated, which matched the design value. Spin-coat 300 nm thick ZEP photoresist and 90 nm thick AR-PC conductive glue on the silicon film to eliminate the influence of poor conductivity of the quartz substrate. The samples were then baked for 2 min to enhance the adhesion of the photoresist. Use EBL to write the graphics area directly on the sample. This process takes about 80 min. Rinse the sample with clean water to remove the conductive glue, and then immediately place it in a developer solution for 100 s to expose the area that needs to be etched. After development, put it into deionized water again for fixing. Employ RIE with CF_4_ and SF_6_ gases to etch the exposed silicon. After etching, the sample was immersed in an acetone solution and heated to 60 °C to eliminate the adhesive. Subsequently, it was washed to achieve the desired metasurface structure.

**Figure 10:**
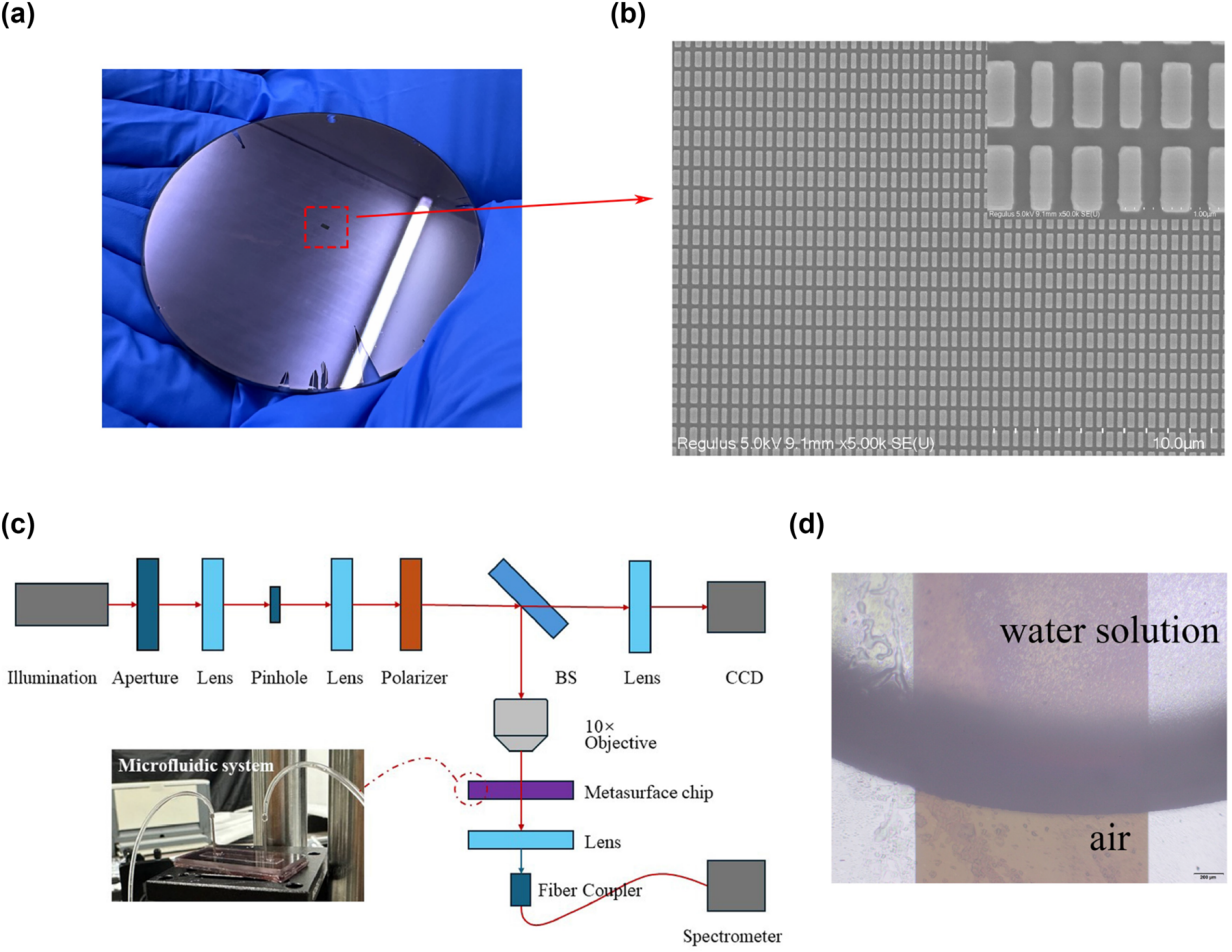
Metasurface fabrication results and testing methods. (a) Metasurface structure of 1 mm × 2  mm area prepared on 3-inch glass substrate. (b) SEM and magnified images of the prepared metasurface. (c) Detection optical path used to detect this metasurface device. (d) Schematic illustration of liquid flowing through the metasurface-microfluidic system.

A depiction of this structure is shown in Figure 10(a). The glass substrate used for this procedure has a dimension of 3 inches. As we use a positive photoresist, the region outside the metasurface is covered by a silicon film of the same height. Meanwhile, Figure 10(b) shows the SEM image and magnified image of the fabricated metasurface, with scale bars of 10 μm and 1 μm. Figure 10(c) shows the optical path used for transmission spectroscopy. The setup utilizes a full spectral range halogen lamp (400 nm–2500 nm) as the light source. After collimation, stray light is filtered out by a pinhole aperture and a rectangular aperture placed at the focus of the lens. A nanoparticle thin film polarizer is used for high extinction ratio (10,000:1) polarization. Subsequently, part of the beam passes through the BS beam splitter and enters the CCD, and part of it is focused on the metasurface through the 10× objective lens. In order to enable convenient testing of the refractive index sensing capabilities of the metasurface sensor, a microfluidic framework is developed and integrated. This particular microfluidic system facilitates the introduction and discharge of fluids via fine needles. The emitted light is connected to the fiber detector of the near-infrared spectrometer via a focusing lens. To ensure stability and optical path accuracy, an air-powered optical platform is used to modify all supporting and optical components.

### 3.2 Experimental sensing performance

**Figure 11:**
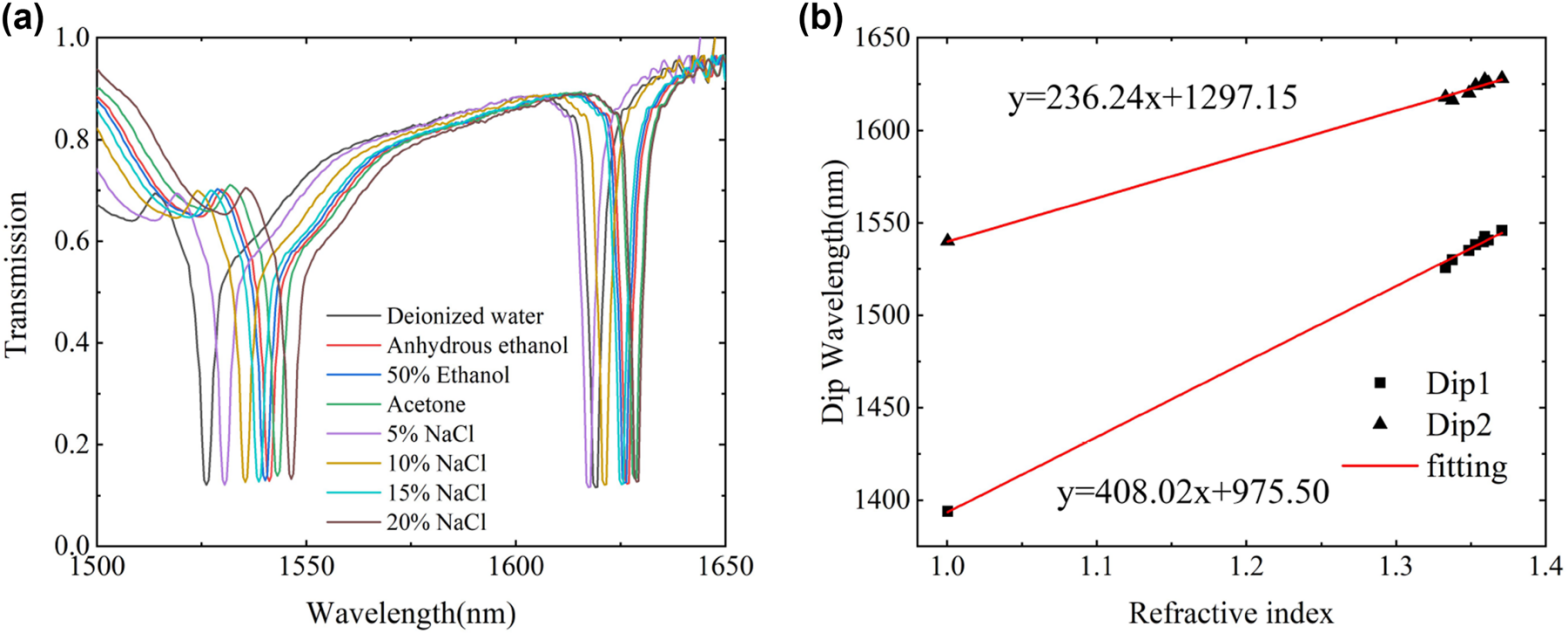
Near-infrared spectroscopy test results of metasurfaces. (a) Designed metasurface transmission spectra after different solutions flow through. (b) The positions of the two resonance peaks and the linear fitting results of the refractive index sensing sensitivity.

Finally, we conducted tests on the sensing performance of the metasurface. The experiment revealed the existence of two separate resonance dips, which exhibited a spectral contrast of over 90 %, and a relatively low resonance linewidth. We tested the transmission spectra of various reagents, and their nominal refractive index values are given in Table 2. The results of the refractive index sensing experiments conducted on the metasurface can be seen in Figure 11(a). The figure shows that the resonance dips undergo a red-shift and the linewidth decreases as the refractive index increases. The two fitted dips have refractive index detection sensitivities of 408.02 nm/RIU and 236.24 nm/RIU, respectively, with FOM values of 107.37 RIU^−1^ and 61.8 RIU^−1^.

**Table 2:** Different types of solutions used in the experimental testing and their nominal refractive index values.

Solute type	Air	Deionized water	Anhydrous ethanol	50 % ethanol	Acetone	5 % NaCl	10 % NaCl	15 % NaCl	20 % NaCl
*n*	1.00027	1.33	1.3618	1.3584	1.3593	1.3377	1.3487	1.3531	1.3707

Table 3 shows the sensitivity, FOM and other performance parameters of the same type of metasurface refractive index sensors. It can be seen that we have achieved dual-channel sensing while ensuring superior sensing performance, which provides an important reference for the development of metasurface devices based on dielectric materials (especially silicon-based).

**Table 3:** Comparison of the experimental performance of this metasurface sensor with the results of other similar structures.

Reference	Materials	Q-factor	*S* (nm/RIU)	FOM(RIU^−1^)
[45]	Si/TiO_2_	728	161.5	78
[48]	Si	–	323	5.4
[49]	Si_3_N_4_	2 × 10^3^	178	445
[50]	Si	23.57^a^	122	4.36^a^
[51]	Si	55^a^	326	21.39^a^
This work (exp.)	Si	365, 397	408, 236	107, 62

The data marked with ^a^are not given in the article and are calculated from other results.

### 3.3 Experimental error analysis

Nonetheless, certain aspects of the experimental results do not fully align with the theoretical simulations, including a wider resonance linewidth and lower spectral contrast (non-zero transmittance at the resonance position). This could be influenced by multiple factors, including fabrication errors of the metasurface, illumination conditions (incident angle, polarization angle, light intensity uniformity, etc.), as well as the matching between experimental materials and simulations and errors introduced by other environmental factors.

The impact of manufacturing errors in metasurface can be seen to follow certain patterns from the analysis in Section 2.2. In the simulations here, we utilized periodic boundary conditions. For a metasurface structure within a certain period, manufacturing errors may lead to a shift in the transmission peak and a decrease in energy in the transmission spectrum. As for large area metasurface devices with a high number of periods, the sum of manufacturing errors will result in broadened linewidths and decreased spectral contrast. In other words, the material properties and metasurface spectral detection methods used in the laboratory (especially the characteristics of the light source) have a more obvious impact on the experimental results.

**Figure 12:**
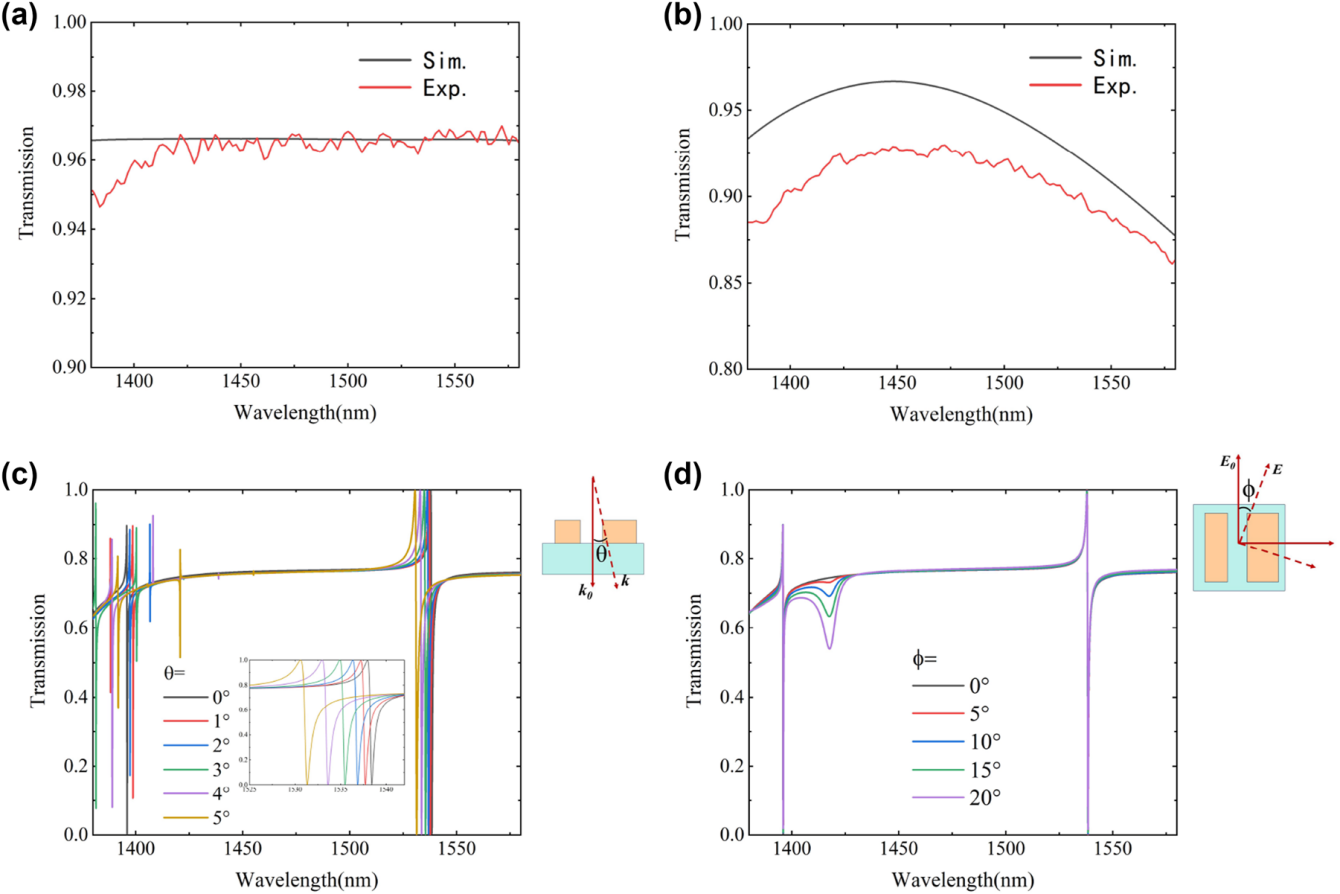
Material properties and error analysis results of metasurfaces. (a) Transmission spectrum of the quartz substrate used. (b) Transmission spectrum of Si after coating. (c) Effect of incident light tilt angle *θ* on metasurface transmission spectrum. (d) The effect of the angle *ϕ* between the polarization direction of the incident light and the *Y* axis on the metasurface transmission spectrum.

An ideal quartz substrate should have a high transmittance. The thickness of the quartz glass used is 1.5 mm, which is much larger than the local field range mentioned above. Figure 12(a) illustrates the quartz substrate’s simulated and experimentally measured transmittance, highlighting excellent agreement between theory and practice. We also analyzed the spectral curves of the Si film, as depicted in Figure 12(b). In the simulation, the Si nanocolumns are 200 nm, but due to manufacturing constraints of the experimental equipment, we first used a dummy wafer to measure the sputtered Si film thickness. After deposition, the silicon thickness was measured using an ellipsometer, which yielded a value of 201.8 nm with an error margin of 0.9 %.

The article employs a halogen lamp as the light source. While the lamp offers full spectrum coverage and user-friendliness, it nevertheless bears some drawbacks. Initially, the halogen lamp’s emission end light source does not constitute an ideal point source; instead, it is a surface light source. This implies that ideal incident conditions for collimation and focusing cannot be realised. To address this, the metasurface was prepared across the largest possible area to minimize its impact. Furthermore, when illuminating the metasurface using a microscope objective, the incident light is not entirely parallel. In some of the edge regions, there exists an angle *θ* between the wave vector and the normal direction, which is discussed in Figure 12(c).

Figure 12(d) simulates the effect of changes in the incident polarization angle *ϕ* relative to the normal direction of the *Y* axis on the transmission spectrum of the metasurface structure. The results indicate that there is no significant shift in the spectral response of the structure, demonstrating a degree of polarization insensitivity.

Moreover, the accuracy errors in data processing and fitting and the spectrometer and CCD themselves, can also have a certain impact on the test results. By optimizing the experimental design and test conditions, it may be possible to reduce the influence of these adverse factors, further improving the practicality and reliability of metasurface sensors.

## 4 Conclusions

In summary, we have devised a metasurface sensor driven by a bound state in the continuum (BIC) that relies on asymmetric double nanorods. Through the regulation of the widths of the various nanorods, the localized BIC is transformed into q-BIC, and two Fano resonances with ultra-high Q factors are excited in the near-infrared band (1400–1700 nm). The resonant frequency and linewidth can be altered by modifying the nanorods’ asymmetric parameters. Electromagnetic field calculations and multipole decomposition indicate that the main mode excited for this resonance is the magnetic dipole (MD) mode. The sensitivity of tests using near-infrared spectroscopy is 408.02 nm/RIU and 236.24 nm/RIU, with a figure of merit of 107.37 RIU^−1^ and 61.8 RIU^−1^. The system achieves dual detection channels without compromising high detection performance. We believe that the sensor proposed in this work exhibits superior performance, high process compatibility, and can play an important role in environmental monitoring, biochemical sensing, and other fields.
